# Molecular dynamics simulation of extraction of Curcuma longa L. extract using subcritical water

**DOI:** 10.1038/s41598-024-79582-x

**Published:** 2024-11-13

**Authors:** Motahareh Gazmeh, Maryam Khajenoori, Sadegh Yousefi-Nasab, Ali Haghighi Asl

**Affiliations:** 1 Faculty of Chemical, Petroleum and Gas Engineering, Semnan University, Semnan, 3513119111 Iran; 2 Materials and Nuclear Fuel Research School, Nuclear Science and Technology Research Institute, Tehran, Iran

**Keywords:** Extraction, Curcuma longa L., Subcritical water, Molecular dynamics simulation, Chemical engineering, Chemical safety

## Abstract

Humans have utilized plants for various purposes, including sustenance and medical treatment for millennia. Researchers have extensively investigated medicinal plants’ potential in drug development, spurred by their rich array of chemical compounds. Curcumin, a valuable bioactive compound, is extracted from Turmeric, known by the scientific name Curcuma Longa L. Notably, curcumin boasts potent antioxidant and anti-inflammatory properties, making it a promising candidate for treating cancer and other microbial diseases. Therefore, the simulation study of the extraction of this important medicinal compound by water, which is a green solvent, was carried out. This study employed molecular dynamics simulation for the first time to explore the extraction of Curcuma Longa L. extract using subcritical water. The simulations were carried out at constant pressure and different temperatures, using the Compass force field in the Lammps simulation package. The findings revealed an increase in the amount of Curcuma longa extract with rising temperature, indicating a weakening of hydrogen bonds in water molecules. Water lost its polar state with increasing temperature and became a suitable non-polar solvent for extracting non-polar compounds. The average absolute relative deviation (AARD) for calculated and simulated density data was 6.45%.

Curcuma longa, a highly prized medicinal plant, holds a significant position, with India being its primary global producer. Traditionally utilized for both its spice and food coloring properties^1^, this plant stands out for its chemical compounds, which play a crucial role in the development of novel drugs^2,3^. Plants contain active chemical compounds such as alkaloids, steroids, phenols, flavonoids, tannins, glycosides, volatile oils, resins, etc. in different parts of the plant such as leaves, flowers, fruits, roots, etc. accumulate^4^. Turmeric, whose scientific name is Curcuma Langa L., is one of the oldest medicinal plants^1^. Buddhists used Curcuma longa as medicine more than 2000 years ago. The Chinese have been using Curcuma longa as medicine for at least 1000 years^5^. Because Curcuma longa has strong antioxidant properties, it is considered one of the most effective anti-cancer substances^6^. Extensive studies show that this plant reduces blood sugar in diabetic patients by increasing insulin secretion, and also reduces blood cholesterol^7,8^. Curcuma longa extract has many biological properties, from cancer control to infections caused by various pathogenic microorganisms. Research indicates that Curcuma longa extract boasts a spectrum of health benefits, encompassing anti-cancer, anti-inflammatory, anti-fungal, anti-microbial, antioxidant, and anti-diabetic properties^9–14^. This yellow crystalline polyphenol, initially isolated from the Curcuma longa plant’s root by Vogel in 1842^5^, has also proven effective in addressing conditions like AIDS, Alzheimer’s, and skin diseases^11,15^. It has been orally consumed for centuries in some regions, with doses reaching 100 mg per day, affirming its long-standing safety for humans^16^. Given that over 90% of the Curcuma longa extract is excreted from the body, researchers are actively exploring ways to boost the bioavailability of this potent plant compound^17^. The absence of defined treatment protocols underscores the ongoing quest to leverage the compound’s potential effectively. The effectiveness of studies, whether quantitative or qualitative, examining Curcuma longa extract relies heavily on the meticulous choice of suitable extraction methods.

Extraction is the first and most basic step for obtaining plant extracts and separating and purifying the chemical components in plants^18^. Extraction (solid-liquid) is defined as the transfer of soluble substances from the solid network to the solvent^19^.

There are various methods for extracting medicinal plants, among which include the Soxhlet method, submerged method, or modern methods such as extraction with supercritical fluid, subcritical water, ultrasound, and microwave^20^. Using organic solvents in the extraction of biologically active compounds from various plants, including Curcuma longa L., is a common method, but it diminishes the extract’s purity. This approach involves substantial solvent quantities, high operating temperatures, and extended time, contributing to environmental pollution^21^. Water, being cost-effective, non-flammable, safe, readily available, non-toxic, and environmentally friendly, emerges as an attractive alternative. Water in the temperature range of 90 °C to 374 °C, so that its pressure is sufficient to maintain the liquid state, is called subcritical water^22^. Subcritical water, or dense hot water, stands out as an effective solvent for both polar and non-polar compounds, representing a novel and safe technology for natural materials^23^. As temperature rises, water’s polarity, surface tension, and viscosity decrease, enhancing solute solubility, diffusion rate, and solvent penetration into the solute matrix^24^. In the process of solvent extraction, there are three basic steps: 1- solvent penetration from the mass in the network, 2- solubility of the compound in the solvent in the network, and 3- re-infiltration of the dissolved compound towards the solvent mass, which depends on the type of extraction and the network. One of these steps will be the process controller. When the solubility of the compound is low, it can be said that it controls the extraction process and requires that the best conditions with the highest solubility are selected for the extraction process^23^. Understanding the solubility of a substance is a crucial attribute that plays a pivotal role in designing, improving, and assessing the viability of separation processes, particularly extraction. The solubility of a solute in an extraction solvent is a key indicator of the extraction process’s efficiency.

The extensive temperature and pressure conditions required for the extraction of medicinal substances give simulation a special place. Computer simulation can be defined as a virtual laboratory. Using molecular dynamics (MD) simulation is a powerful tool that models molecules and generates information at the microscopic level^25,26^. In 2016, Anisa and colleagues employed molecular dynamics simulation to assess the permeation rate of bioactive compounds in ginger under subcritical water and ethanol conditions. Their findings indicated that as the ethanol percentage and temperature rose, so did the permeation coefficients^27^. Similarly, in 2019, Li Zhang and team investigated the interaction between Curcuma longa L extract and myosin molecules through molecular dynamics simulation^28^. In our previous work in 2023, the solubility parameter of pure curcumin in subcritical water using experimental methods, and molecular dynamics simulations was calculated^29^. It is believed that molecular dynamics simulation can describe the extraction behavior of curcumin extract from Curcuma Longa L.

Considering the use of Curcuma longa L. throughout history for home use and treatment of diseases, as well as the current medical use of this valuable antioxidant compound, research was done on it^30,31^. Despite extensive research on Curcuma longa L., there are still important gaps such as its low bioavailability. To increase the bioavailability of Curcuma longa L., it is emphasized that the extract be extracted using appropriate methods. Therefore, this research has tried to simulate the extraction of Curcuma longa L. extract by molecular dynamics method. To explore the interaction between curcuma longa L. extract and subcritical water across diverse temperatures, molecular dynamics simulations were innovatively employed for the first time. These simulations, maintained under a constant pressure of 2 MPa, spanned temperatures from 363.15 to 453.15 K. Utilizing the Compass force field and Velocity Verlet movement algorithm within the LAMMPS simulation package, the study delved into mean square displacement (MSD) and radial distribution function (RDF) charts at different temperatures. The objective was to discern the influence of temperature on solubility and the intermolecular dynamics between solute and solvent.

## Simulation method

**Fig. 1:**
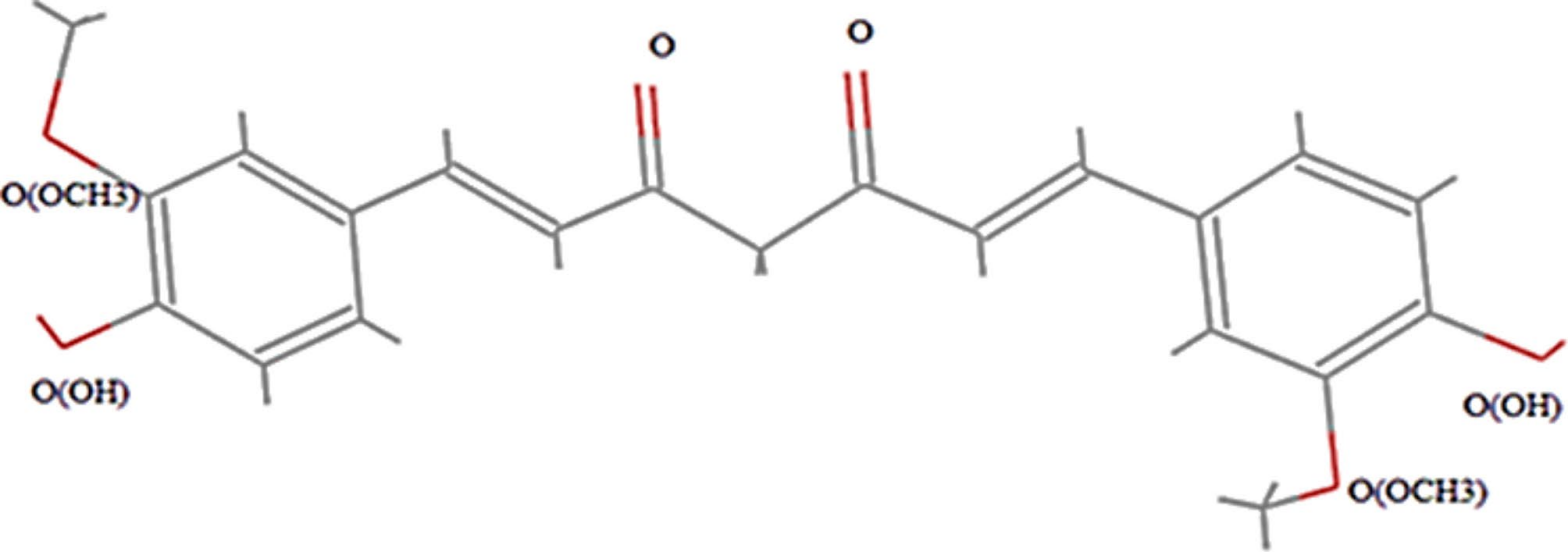
Molecular structure of curcumin.

In this investigation, molecular dynamics simulations were utilized to explore the extraction of Curcuma longa L. plant extract using subcritical water. Employing the Lammps software package alongside the Compass force field and Velocity Verlet movement algorithm, simulations were performed across a range of temperatures. Curcumin, which constitutes over 90% of Curcuma longa L. extract^32,33^, as illustrated in Fig. 1, was considered as the model compound while subcritical water served as a solvent.

One molecule of curcumin and 300 molecules of water make up the system, employing force field parameters derived from Lammps, within a 21.2 × 21.2 × 21.2 angstroms box with random molecule dispersion and periodic boundary conditions. System equilibrium was achieved through a 1 nanosecond NVT simulation, transitioning molecules from a random to a stable state. Subsequent molecular dynamics simulations under NPT conditions utilized the Nose-Hoover thermostat and Berendsen barostat at fixed temperatures (363.15, 393.15, 413.15, 423.15, and 453.15 K) with a constant one femtosecond time step. Leonard Jones and electrostatic forces were governed by a 12.5 Angstroms cut-off radius. The simulations ran for 5 nanoseconds to attain equilibrium and an additional 1 nanosecond for data collection.

Here, considering that the solubility of Curcuma longa L. in subcritical water is very low, the solubility of this compound controls the extraction process. To ascertain the solubility of a solute in a solvent, it becomes essential to compute the free energy parameter of solubility. This parameter, known as the free energy of solvation, is characterized as the reversible work needed to relocate a solute molecule from a fixed position in an ideal gas to a fixed position within a solution. In employing the MD simulation method to calculate this free energy, one must systematically assess the evolving interaction between a singular solute molecule and the remainder of the solute in NPT or NVT groups^34,35^. The interplay between solubility and solubility free energy finds elucidation through the application of a thermodynamic equation (Eq. 1)^36^:

1 $$y_{2} ~ = \frac{{P_{2}^{{sat}} \exp \left[ {\beta \vartheta ^{s} \left( {P - P_{2}^{{sat}} } \right)} \right]}}{{\rho k_{B} Texp\left( {\beta \Delta G^{{solv}} } \right)}}$$

In Eq. 1, $\:{y}_{2}$ is the mole fraction of the solute and ρ is the density of the pure solvent. In this equation, β = 1/$\:{k}_{B}$T, where $\:{k}_{B}$ and T are the Boltzmann constant and the temperature of the furnace, respectively. Also, $\:{P}_{2}^{sat}$ and $\:{\vartheta\:}^{s}$ are respectively sublimation pressure and molar volume of a specific substance.

To assess the extent of substance penetration or diffusion into another material, one can examine the penetration coefficient. Penetration, essentially the displacement of mass through atomic transfer, involves the movement of atoms causing a shift in matter. In fluid environments, such as liquids and gases, penetration is driven by the random Brownian motion resulting from the unpredictable movement of particles within the fluid^37^. The molecular diffusion coefficient (D), measured in m²/s, serves as a temperature, pressure, and material-dependent physical property. In MSD analysis, Einstein’s equation^38^ allows the determination of the molecular diffusion coefficient by calculating the slope of the MSD graph against time (t).:

2 $${\text{MSD}} = 6{\text{Dt}} + {\text{c}}$$

An increase in temperature affects the rate of mass transfer by increasing molecular diffusion and causes higher penetration, which leads to an increase in extract extraction efficiency^39,40^. To gain more knowledge about the extraction of Curcuma longa L. extract in subcritical water, the diffusion coefficient values ​​of Curcuma longa L. extract were simulated, which are discussed in the next section.

Moreover, for a detailed exploration of materials at the molecular level and the analysis of their intermolecular interactions, it is insightful to delve into the outcomes of Radial Distribution Functions (RDF). The RDF stands as a prevalent physical parameter in molecular dynamics simulations. In the context of each mixture, multiple radial distribution functions might be pertinent. The RDF, denoted as g(r), provides insight into the likelihood of locating a fluid atom at a certain distance like ‘r’ from other fluid atoms, and its definition is encapsulated in Eq. 3^41^:

3 $$g\left( r \right)~ = \frac{{Ny\left( {r,~r~ + ~dr} \right)~}}{{\rho _{y} 4\pi r^{2} dr}}$$

In Eq. 3, r is the radius of the sphere, $\:{\rho\:}_{y}$ is the atom density and $\:\text{N}\text{y}(\text{r},\:\text{r}+\text{d}\text{r})$ is a function that calculates the number of fluid atoms in a shell with a thickness of dr. In this study, the intermolecular interaction in the binary mixture of Curcuma longa L. and subcritical water was investigated.

## Results and discussion

### Solubility

**Fig. 2:**
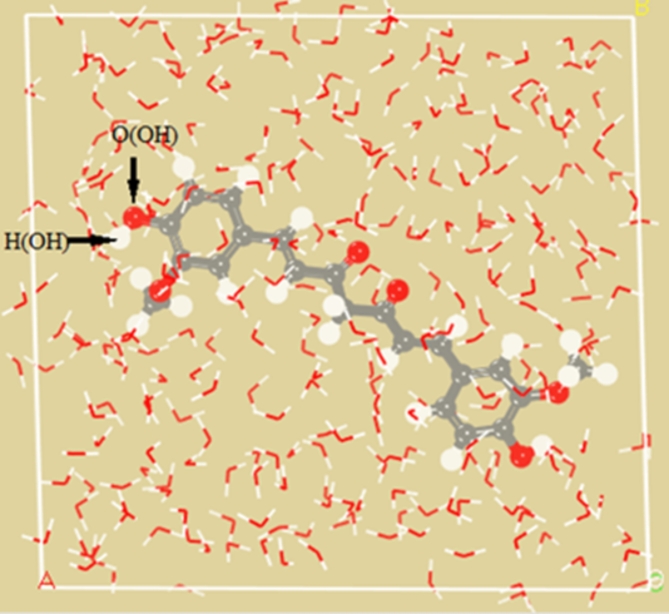
The simulated box of curcumin molecules and water at a temperature of 363.15 K and a pressure of 2 MPa. Color display of atoms: gray: carbon, red: oxygen, and white: hydrogen.

Solubility serves as a key factor delineating the thermodynamic boundaries of a process, exerting a pivotal influence on the economic efficiency of said process. Data on solubility offer crucial insights into extraction time, yield, and the optimal extraction temperature. Consequently, the quantification of solubility stands as a paramount parameter for achieving optimal extraction under specific operating conditions. It is defined as the maximum amount of solute that can dissolve in a given quantity of solvent at a particular temperature and pressure^42,43^. To ascertain the solubility parameter, the free energy of solubility was calculated at various temperatures through molecular dynamics simulation, as detailed in Table 1. Figure 2 illustrates the simulation box encompassing soluble and solvent molecules, simulated at 363.15 K and 2 MPa pressure.

**Table 1:** Data on solvation free energy across various temperatures and a pressure of 2 MPa.

Temperature (K)	Solvation free energy (Kj.mol^− 1^)
363.15	-35.848
393.15	-35.246
413.15	-35.600
423.15	-35.400
453.15	-35.590

Curcumin possesses a molar mass of 3.961 × $$\:{10}^{-4}$$$$\:{m}^{3}$$.mol^− 1^^44^, and its vapor pressure at 25 degrees Celsius is 3.08 × $$\:{10}^{-12}$$ mmHg^45^. The sublimation pressure of curcumin at various temperatures was calculated using the Clausius-Clapeyron equation^46^. In addition, the density of subcritical water at different temperatures was determined based on the literature^24^. The simulated solubility data of Curcuma longa L. extract in subcritical water calculated using Eq. 1 are presented in Table 2. In our previous work in 2016, Curcuma longa extract was extracted with subcritical water solvent^21^. Experiments were performed on the powder obtained from the fresh rhizomes of Curcuma longa of Indian origin purchased from a local market (Shahmirzad, Semnan, Iran) using the dynamic method in the temperature range of 363.15 to 423.15 K, the water flow rate was 1-4 ml.$$\:{min}^{-1}$$ and the average particle size was 0.5 to 1.5 mm. The effect of all three parameters, temperature, water flow rate, and particle size, was investigated simultaneously on the extraction efficiency. The results showed that the optimal conditions for extracting curcumin from the rhizome of Curcuma longa are at a temperature of 423.15 K, with a water flow rate of 1 mm/min and a particle size of 0.5 mm. It was also found that increasing the temperature increases the extraction efficiency. The solubility parameters obtained from both methods are presented in Table 2 to compare the simulation results with the experimental results. According, to the obtained results, it can be seen that molecular dynamics simulation can well predict the extraction behavior of Curcuma longa extract with increasing temperature.

**Table 2:** Simulated solubility data for Curcuma longa L. extract in subcritical water, examined across varying temperatures and under a pressure of 2 MPa.

Temperature (K)	Simulated density [gr.cm^− 3^]	Calculated density [gr.cm^− 3^]	Simulated Solubility×10^6^	Experimented Solubility×$\:{10}^{6}$^21^
363.15	0.902	0.962	3.3	1.39
393.15	0.896	0.946	10.2	13.3
413.15	0.874	0.935	13.0	18.8
423.15	0.855	0.929	33.0	–
453.15	0.781	–	120	–

Observing the data, it’s evident that the extraction efficiency of Curcuma longa L. extract escalates with rising temperatures, attributed to the altered properties of water as a solvent. With increasing temperature, water’s dielectric constant decreases, transforming its behavior to mimic organic solvents capable of dissolving both polar and non-polar substances. Notably, the highest solubility of Curcuma longa L. extract occurs at 453.15 K; however, it’s crucial to acknowledge that excessive temperature increases might jeopardize the structure of organic compounds, a limitation inherent to the simulation software. To validate the simulation, the Absolute Average Relative Deviation (AARD) was computed, yielding a favorable result of 6.45% for density data. This affirms the reliability of the simulation outcomes.

## Exploring the impact of temperature on diffusion coefficient

**Fig. 3:**
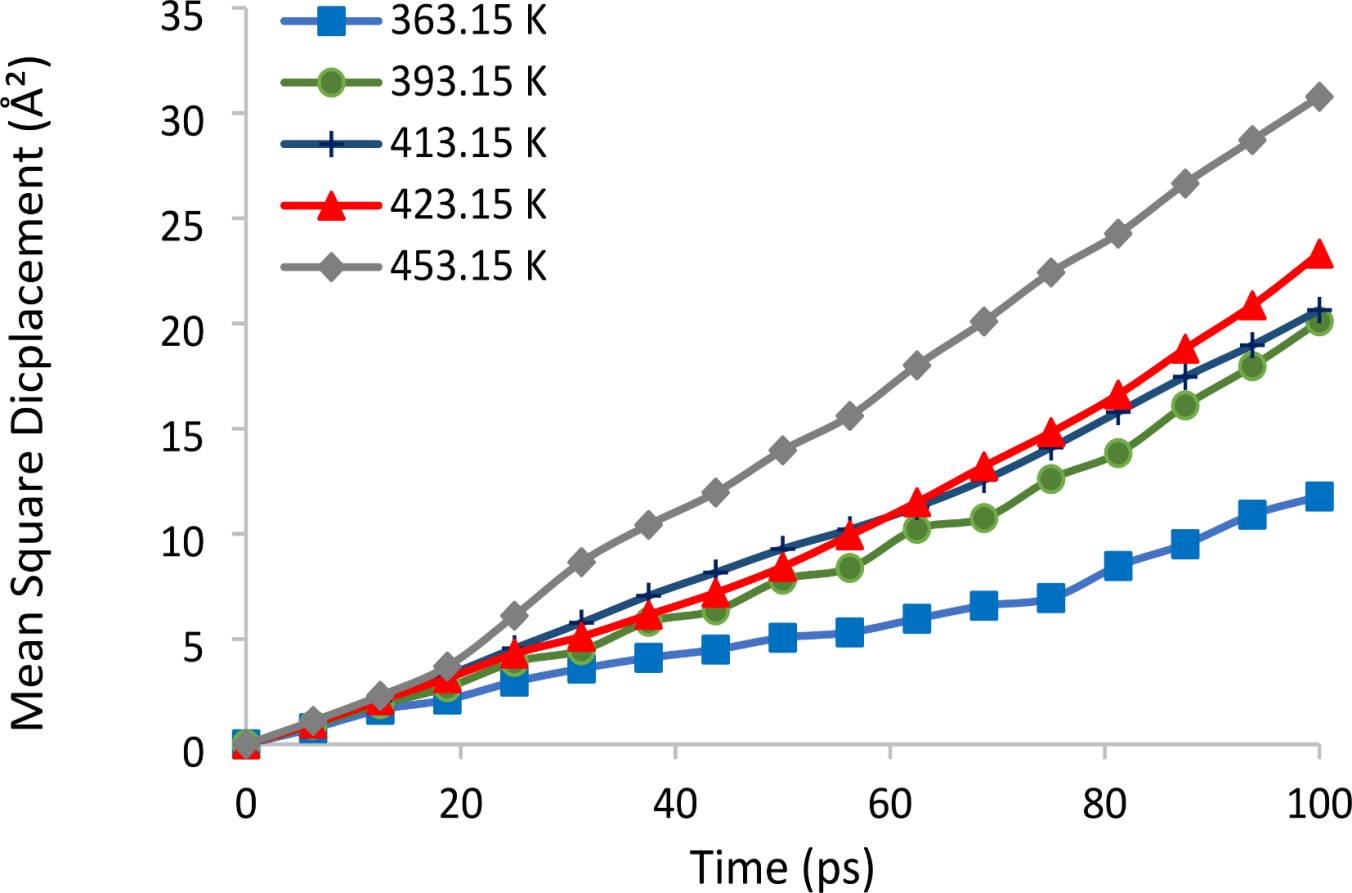
Diagram of mean square displacement of Curcuma longa L. extract at different temperatures.

To ascertain the penetration coefficients of Curcuma longa L. extract, MSD diagrams were generated across various temperatures (Fig. 3). Notably, the slope of the MSD diagram rises proportionally with temperature increments, a consequence of heightened molecular kinetic energy. The observed increase in mean square displacement over time across all five temperatures indicates an augmented penetration rate of curcumin in subcritical water with rising temperature. Consequently, the solubility of curcumin in subcritical water experiences an increase with increasing temperature. Table 3 presents the calculated diffusion coefficients of curcumin at different temperatures.

**Table 3:** The values ​​of the diffusion coefficient of Curcuma longa L. extract in subcritical water at different temperatures.

Temperature (K)	D [m^2^.s^− 1^] ×10^9^
363.15	4.4
393.15	8.1
413.15	8.5
423.15	9.6
453.15	11.2

## Impact of temperature on radial distribution functions

Each mixture may exhibit multiple radial distribution functions, and this study delves into functions pertinent to hydrogen bonding. To validate the simulation initially, pure water was simulated using the COMPASS force field. The radial distribution function (RDF) diagrams, specifically focusing on the bond between pure water molecules, were carefully examined. was revealed that the first peaks associated with oxygen-hydrogen, hydrogen-hydrogen, and oxygen-oxygen bonds are located at 1.75 Å, 2.25 Å, and 2.75 Å, respectively. A comparative analysis with the findings of Hashim et al.^47^ demonstrated a noteworthy correlation, affirming the reliability of the simulation. These results underscore the significance of the O-H bond as the nearest neighbor interaction, surpassing the strengths of O-O and H-H bonds, indicative of the hydrogen bonding among water molecules.

**Fig. 4:**
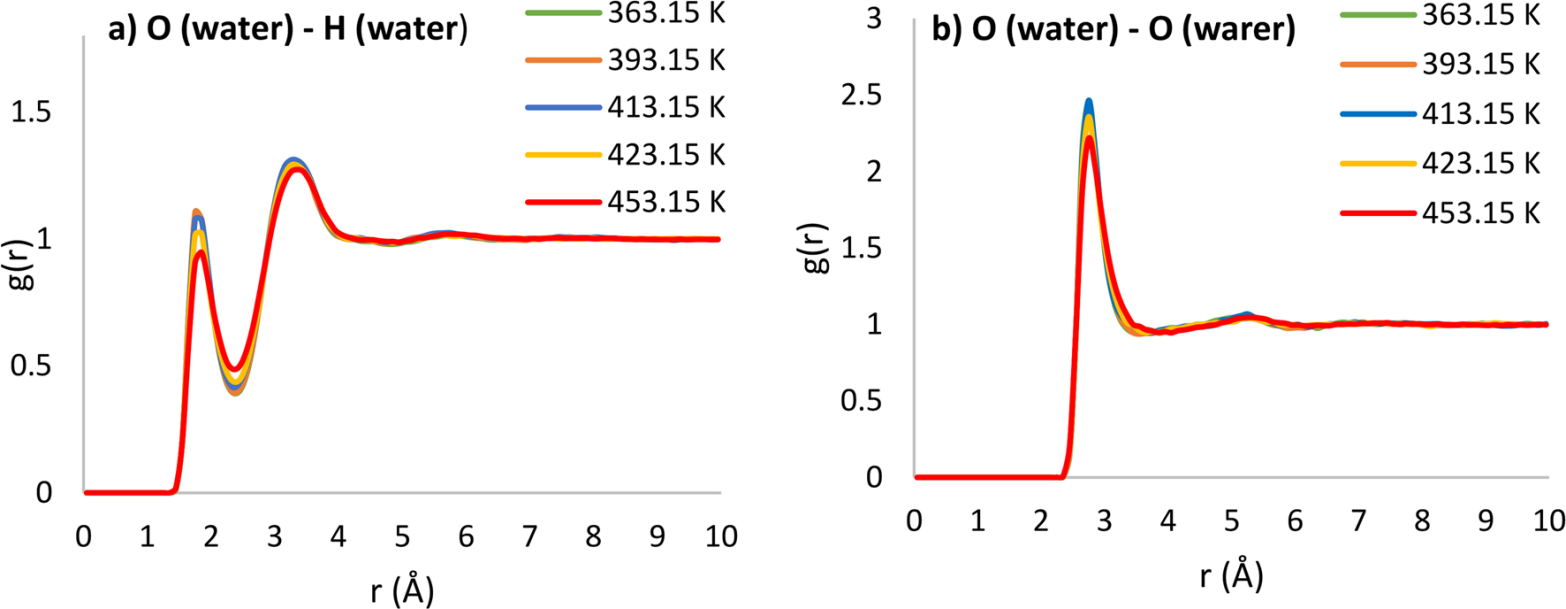
Comparison of interstitial interaction between water molecules at different temperatures for (**a**) O(water)-H(water) and (**b**) O(water)-O(water).

Upon establishing the robust validity of the simulation, subsequent simulations were executed for both water and Curcuma longa L. solution. These simulations delved into the analysis and investigation of intermolecular interactions between solvent-solvent and solute-solvent. Figure 4 illustrates the intermolecular interactions among water molecules within the two-component system of curcumin and subcritical water at varying simulation temperatures.

**Fig. 5:**
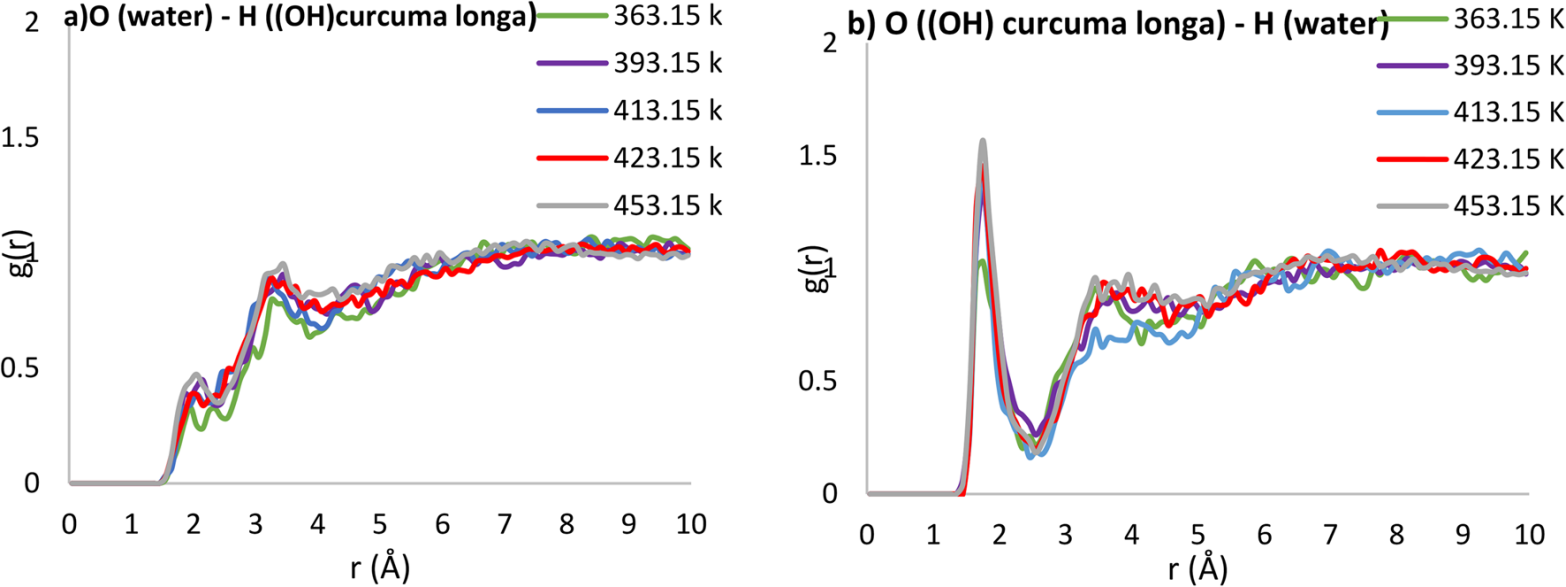
illustrates the comparative analysis of intermolecular interactions between solute and solvent molecules at various simulation temperatures for both (**a**) and (**b**).

In Fig. 4, the initial peak corresponding to oxygen(water)-hydrogen(water) and oxygen(water)-oxygen(water) interactions is depicted at distinct temperatures, occurring at radial distances of 1.75 Å and 2.75 Å, respectively. This provides insight into the strength of hydrogen bonding among water molecules in the system. Notably, as the temperature rises, the intensity of these peaks diminishes. For instance, the intensity of the oxygen(water)-hydrogen(water) interaction at a distance of 1.75 angstroms exhibits values of 1.10, 1.08, 1.07, 1.01, and 0.90 at temperatures of 363.15, 393.15, 413.15, 423.15, and 453.15 K, respectively, indicating a decline in hydrogen bonding strength with increasing temperature. The intensity of peaks in the RDF structure of a two-component system serves as an indicator of a robust bond between the involved components. Figure 5 illustrates the interaction between solute and solvent at diverse simulation temperatures.

In Fig. 5a, the nearest peak corresponding to O(water)-H(OHcurcumin) at a radial distance of 3.25 Å is illustrated, displaying values of 0.78, 0.81, 0.87, 0.93, and 0.98 for simulated temperatures of 363.15, 393.15, 413.15, 423.15, and 453.15 K, respectively. Notably, as the temperature rises, the intensity of this peak increases, signifying an augmentation in the extraction efficiency of Curcuma longa L. extract in water. Moving to Fig. 5b, the O(OHcurcumin) H(water) interaction exhibits noteworthy intensity at a distance of 1.75 Å. The heightened intensity in the O(OHcurcumin)-H(water) interaction implies that the bond between the oxygen atom of curcumin and the hydrogen atom of the water molecule is more robust than other bonds. This particular bond serves as a key communication factor between the introduced Curcuma longa L. and water.

Table 4 provides a summary of the interaction intensities between Curcuma longa L. and subcritical water, offering insights into the bonding tendencies between solute and solvent molecules. For instance, at 363.15 K, the bond intensity between the first neighboring atoms at a distance of 1.75 angstroms was 1.03. Notably, as the temperature rises, the interaction intensity between curcumin molecules and subcritical water augments, forming larger peaks and indicating an enhanced extraction efficiency. This observation is consistent with the previous findings in Tables 2 and 3. The rationale behind this lies in the increased temperature facilitating solute diffusion, while concurrently weakening the hydrogen bonds in water, reducing its polarity and rendering it a more favorable solvent. Consequently, the likelihood of bonding between solvent and solute atoms increases.

**Table 4:** The intensity of interaction between Curcuma longa L. extract and subcritical water at different temperatures.

Temperature (K)	Curcumin- subcritical water interaction	peak intensity
363.15	O(OH curcumin)-H(water)	1.03
393.15	O(OH curcumin)-H(water)	1.34
413.15	O(OH curcumin)-H(water)	1.42
423.15	O(OH curcumin)-H(water)	1.45
453.15	O(OH curcumin)-H(water)	1.56

## Conclusion

Herbal medicines have a special place and the extraction of their extracts by green solvents is a very important issue that has attracted the attention of scientists. Different methods are used to extract the extract of medicines. Most of these methods have limitations and are not used in a wide range of temperatures and pressures. Recently, modeling approaches, particularly molecular dynamics simulation, have gained prominence for their accuracy. In this study, the extraction of Curcuma longa L. extract by subcritical water was investigated using molecular dynamics simulation and COMPASS force field. The free energy of solubility was used to predict the extraction of Curcuma longa L. extract in subcritical water. Also, the amount of penetration of Curcuma longa L. extract in subcritical water was studied and discussed through MSD diagrams and solute and solvent intermolecular interactions through RDF diagrams. A comprehensive examination from various perspectives revealed that as the temperature increased, the extraction rate of Curcuma longa L. extract also increased. Higher temperatures not only elevated the penetration coefficient of the extract in subcritical water but also weakened the strength of hydrogen bonding among solvent molecules. Conversely, at elevated temperatures, the strength of the hydrogen bonds formed between the solute and the solvent increased. The interaction between atoms of O(OH-curcumin) and H(water) was identified as a key bonding factor between Curcuma longa L. and subcritical water. This study underscores the significance of molecular dynamics simulation in elucidating the complex dynamics of herbal extract extraction processes.

## Data Availability

All data generated or analysed during this study are included in this published article.
